# Quantitative, Architectural Analysis of Immune Cell Subsets in Tumor-Draining Lymph Nodes from Breast Cancer Patients and Healthy Lymph Nodes

**DOI:** 10.1371/journal.pone.0012420

**Published:** 2010-08-25

**Authors:** A. Francesca Setiadi, Nelson C. Ray, Holbrook E. Kohrt, Adam Kapelner, Valeria Carcamo-Cavazos, Edina B. Levic, Sina Yadegarynia, Chris M. van der Loos, Erich J. Schwartz, Susan Holmes, Peter P. Lee

**Affiliations:** 1 Department of Medicine, Stanford University School of Medicine, Stanford, California, United States of America; 2 Department of Statistics, Stanford University, Stanford, California, United States of America; 3 Department of Pathology, Academic Medical Center, Amsterdam, The Netherlands; 4 Department of Pathology, Stanford University School of Medicine, Stanford, California, United States of America; Institut Pasteur, France

## Abstract

**Background:**

To date, pathological examination of specimens remains largely qualitative. Quantitative measures of tissue spatial features are generally not captured. To gain additional mechanistic and prognostic insights, a need for quantitative architectural analysis arises in studying immune cell-cancer interactions within the tumor microenvironment and tumor-draining lymph nodes (TDLNs).

**Methodology/Principal Findings:**

We present a novel, quantitative image analysis approach incorporating 1) multi-color tissue staining, 2) high-resolution, automated whole-section imaging, 3) custom image analysis software that identifies cell types and locations, and 4) spatial statistical analysis. As a proof of concept, we applied this approach to study the architectural patterns of T and B cells within tumor-draining lymph nodes from breast cancer patients versus healthy lymph nodes. We found that the spatial grouping patterns of T and B cells differed between healthy and breast cancer lymph nodes, and this could be attributed to the lack of B cell localization in the extrafollicular region of the TDLNs.

**Conclusions/Significance:**

Our integrative approach has made quantitative analysis of complex visual data possible. Our results highlight spatial alterations of immune cells within lymph nodes from breast cancer patients as an independent variable from numerical changes. This opens up new areas of investigations in research and medicine. Future application of this approach will lead to a better understanding of immune changes in the tumor microenvironment and TDLNs, and how they affect clinical outcomes.

## Introduction

Medicine is evolving from a qualitative to a quantitative science. This has led to improved diagnostic and prognostic tools as well as novel therapies. Tissue analyses at the DNA, RNA, and protein levels through protein and microarray technologies are examples of these advances. Histological evaluation of patient tissues is a field that would likely benefit from improved quantitative measurement, but currently methods for accurate quantification are not readily available. Spatial relationships between cells may provide additional information of prognostic or therapeutic significance [1]. Qualitative measures of tumor characteristics remain the standard of practice. Spatial measures, such as average distance between cells or local density of cells, are generally overlooked in research or clinical studies, largely due to the laborious nature of manually scoring histological data, generating a quantitative code which captures the histological characteristics, and performing statistical analysis of this data.

Prognosis and treatment for women with breast cancer is dependent upon status of sentinel and non-sentinel axillary lymph nodes (SLNs and ALNs) [2]. Current pathological analysis of tumor-draining lymph nodes (TDLNs) focuses solely on the presence or absence of tumor cells [2], [3]. We previously showed that significant alterations in immune cell populations arise in the ALNs from women with breast cancer [4]. Our findings suggest perturbation of the immune profiles could arise in a tumor cell dependent or independent fashion, as changes in lymphocyte populations developed in lymph nodes that were involved with and also free of infiltrating tumor cells. Importantly, the immune changes observed strongly correlated with clinical outcome. As an extension of these results, we hypothesize that the architectural relationships between immune cell subsets and with infiltrating tumor cells within TDLNs may provide additional mechanistic and prognostic information.

To address this hypothesis, we developed a quantitative image analysis approach. This consists of multicolor staining of tissue sections; high-resolution, automated whole-section imaging; custom image analysis software capable of quantifying cell population size; and analyses of spatial relationships between various cell types within entire tissue sections. We applied this approach to quantify and analyze the architectural patterns of immune cell subsets, specifically T and B lymphocytes as the major constituents of the lymph nodes, within TDLNs from breast cancer patients and healthy lymph nodes (HLNs). In this study we found that even when the proportions of T and B cells are similar, the spatial grouping patterns of these cells differed between healthy and tumor-draining lymph nodes. This technology is a powerful tool to study cell population sizes and spatial patterns between various cell types in tissue sections. These types of analyses can better elucidate the complex relationships between quantitative and spatial cellular information with clinical parameters and patient outcomes.

## Results

### Quantitative image analysis approach

**Figure 1:**
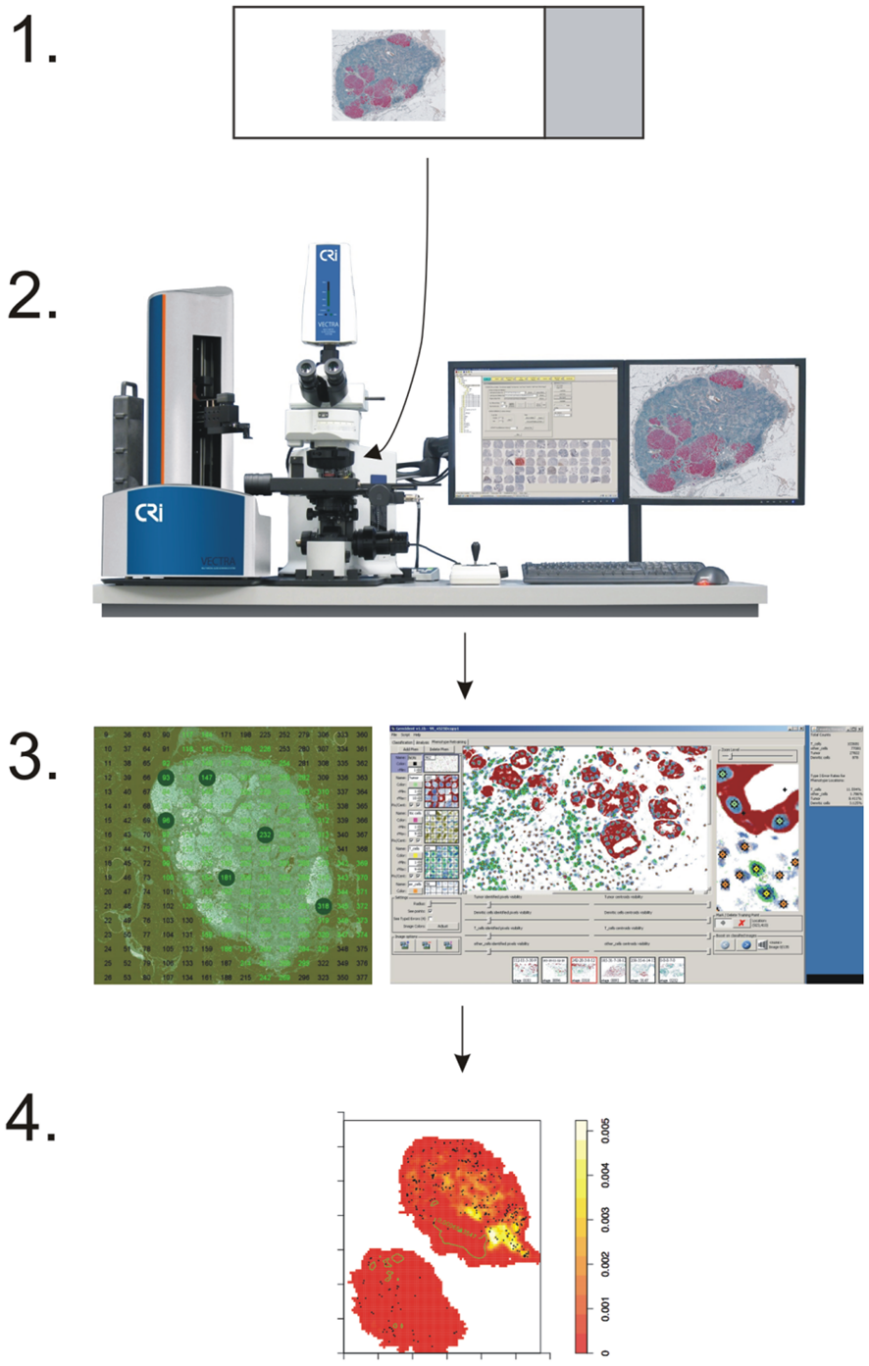
Integrated image analysis approach. Stage 1: multicolor staining of tissue sections. Stage 2: high-resolution spectral imaging and automated scanning of the entire tissue section. Stage 3: machine-learning-based cell identification by GemIdent. Stage 4: numerical and spatial statistical analyses, such as cell densities, distances and distributions.

To analyze the spatial relationships between several different cell populations within tissue, samples were stained using multiple chromogens and the entire tissue sections were imaged at high resolution. An illustration of the integrated image analysis approach is presented in Figure 1. In a 3-color IHC stained tumor-infiltrated lymph node cross section, we could concurrently visualize tumor cells (red), T cells (dark blue) and B cells (brown) (Fig. S1). All nuclei were counter-stained with hematoxylin (light blue). An image of an entire lymph node section consists of hundreds of 200×, high-resolution sub-images, depending on the size of the tissue section. This imaging system unmixes spectra from the various chromogens used and reconstructs the image in pseudo-colors chosen by the researcher (Fig. S2). All 200× images that composed an entire lymph node section were then analyzed using a custom image analysis software, GemIdent, yielding the total number of cells for each stained phenotype and the location (x, y coordinate) of each cell identified [5]. These data, collected via the multi-spectral approach and semi-automated features of the image acquisition and analysis tools, made various spatial statistical analyses possible. Examples of such analyses include the measurement of distances between cells' nuclei, local densities, and the observation of architectural patterns of different cell populations.

### Spatial grouping patterns of T and B cells differed between tumor-draining and healthy lymph nodes

As a proof of concept, we tested our quantitative image analysis approach to assess the relationship between proportions and spatial properties of T and B cells within TDLNs from breast cancer patients. SLNs+ from 15 breast cancer patients (1 SLN per patient), pairs of ALN+ and ALN− from 10 patients, and 7 HLNs from prophylactic mastectomy and breast reduction patients were selected. To allow comparisons of immune cell populations between lymph nodes that have varying degrees of tumor infiltrations, tumor cells were excluded from the calculation of total number of cells in tumor-invaded lymph nodes. Median T cell proportions (±SD) were 54.9% (±13.3%), 49.3% (±13.7%), 45.4% (±9.9%) and 57.7% (±11.7%), for SLN+, ALN+, ALN− and HLN; B cells were 34.2% (±12.6%), 27.2% (±9.7%), 31.2% (±14.1%) and 34.0% (±11.8%), respectively (Fig. S3A and B). From these TDLNs and HLNs that have similar proportions of T and B cells, we first compared the spatial grouping patterns of these two major cell populations against each other. Using the L function method, a variant of Ripley's K function [6], we investigated whether or not cells within the two populations have similar tendency to group with or disperse from each other. We analyzed the T and B cells in tumor-free areas in the TDLN and compared against HLN cross-sections.

As a reference target distribution, we generated the L function from T cell data, which are the locations of all T cells identified in a particular lymph node section. We then tested whether B cells within the same tissue section follow the same grouping pattern as the T cells. A variety of L function plots and their interpretations are illustrated in Figure S4A. The confidence envelope was generated by a Monte Carlo simulation of data from the T cells [7]. If the L function of the B cells falls within the confidence envelope generated from the T cell data, this indicates that the B cells have the same tendency to group at various distances as the T cells within the same tissue section. If the L function of the B cells deflects above the envelope, it indicates that the B cells are more clustered than the T cells. In contrast, if the L function of the B cells deflects below the envelope, it indicates that the B cells are more dispersed than the T cells. Figure S4B illustrates an example that shows an L function of B cells that exits upward, starting at the inter-cell (interpoint) distance of 100 µm and remaining outside of the envelope until an interpoint distance of 450 µm. The interpretation is that for interpoint distances between 100 µm and 450 µm, the B cells exhibit more clustering than the T cells.

**Figure 2:**
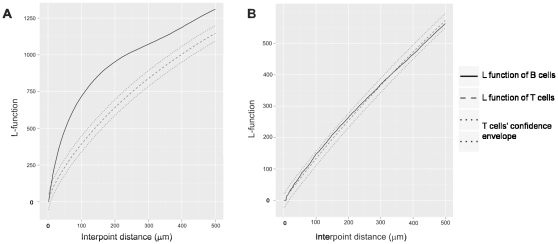
Representative L function plots of T and B cells in TDLNs and HLNs. (A) In most TDLNs, B cells are more clustered than the T cells, as indicated by upward deflection of the L function of B cells above the confidence envelope. (B) In the majority of the HLNs, T and B cells have a similar tendency to group or disperse at various interpoint distances, as indicated by the L function of B cells that remains within the confidence envelope.

In the majority of the TDLNs, B cells' L functions exited the confidence envelopes constructed from the T cells data below the interpoint distance of 100 µm. In all of these cases, the graph deviated above the envelope, indicating that the B cells were more clustered than the T cells. This was observed in 9 out of 10 (90%) of the ALNs+, 6 out of 10 (60%) of the ALNs−, and 8 out of 15 (53%) of the SLNs+. The shorter the interpoint distance at which the B cells' L function exited above the confidence envelope, the more local the B cell clustering was observed relative to the T cells. A representative plot of L function versus interpoint distance up to 500 µm is shown in Figure 2A. In contrast, in the majority of the HLNs, the B cells had a similar tendency to group at various distances with each other, as the T cells within the same tissue section. This was indicated by the B cells' L functions that remained within the confidence envelopes constructed from the T cells data. Below the interpoint distance of 100 µm, this was observed in 4 out of 7 (57%) of the HLNs analyzed. A representative plot that displays the L function of B cells that remains within the T cells' envelope is shown in Figure 2B.

**Figure 3:**
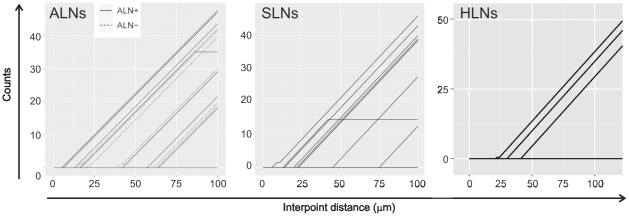
Plots illustrating the number of TDLNs and HLNs in which B cells were found to be more clustered than the T cells. Each line represents data from 1 lymph node. The shorter the interpoint distance at which the B cells' L function exited above the confidence envelope, the more clustered B cells are compared to the T cells. Below the interpoint distance of 100 µm, the number of lines that represent the L function of B cells outside of the T cells' envelope is as follows: 9 out of 10 (90%) ALNs+, 6 out of 10 ALNs−, 8 out of 15 (53%) SLNs+ and 3 out of 7 (43%) HLNs. The counts indicated how much more clustered B cells are compared to T cells, and were determined as follows: we evaluated the L-function of the B cells for interpoint distances of 0 to 100 µm in 2 µm increments and compared it with the confidence envelope of the L-function for the T cells. For each interpoint distance increment of 2 µm for which the L-function of the B cells lay above the envelope, the value of the counts increased by one. The starting point was set at 0.

Next, we plotted lines indicating the number of lymph nodes in which B cells were found to be more clustered than the T cells (Fig. 3). We then determined the average counts spent by the L function of B cells above the T cells' envelope between the interpoint distance of 0 and 20 µm, within which several lines of the B cells' L functions from the TDLNs had started to exit the envelopes, then compared the values between the SLN+, ALN+, ALN− and HLN groups. The higher the count, the larger the difference between spatial grouping patterns of the local T and B cells. The result showed no L function of any HLN deflected away from the T cells' envelope, yielding zero as the count between the interpoint distance 0 and 20 µm. This was followed by the ALN−, SLN+ and ALN+, with the average counts of 0.25, 0.8 and 2.1, respectively. A significant difference was found between the ALN+ and HLN groups (p = 0.02; t-test).

In summary, the spatial grouping patterns of T and B cells differed between TDLNs and HLNs. In most of the TDLNs, B cells were more clustered than T cells. As we focused on a smaller range of interpoint distance, we found that the difference in local T and B cell grouping patterns was the highest in tumor-invaded lymph nodes. However, no correlation was observed between the amount of tumor burden in the lymph nodes analyzed and the similarity or discrepancy between the T and B cell grouping patterns. In contrast, in the majority of the HLNs, B cells similarly grouped at various distances from each other as the T cells within the same tissue section.

### TDLNs and HLNs have different B cell localization patterns

**Figure 4:**
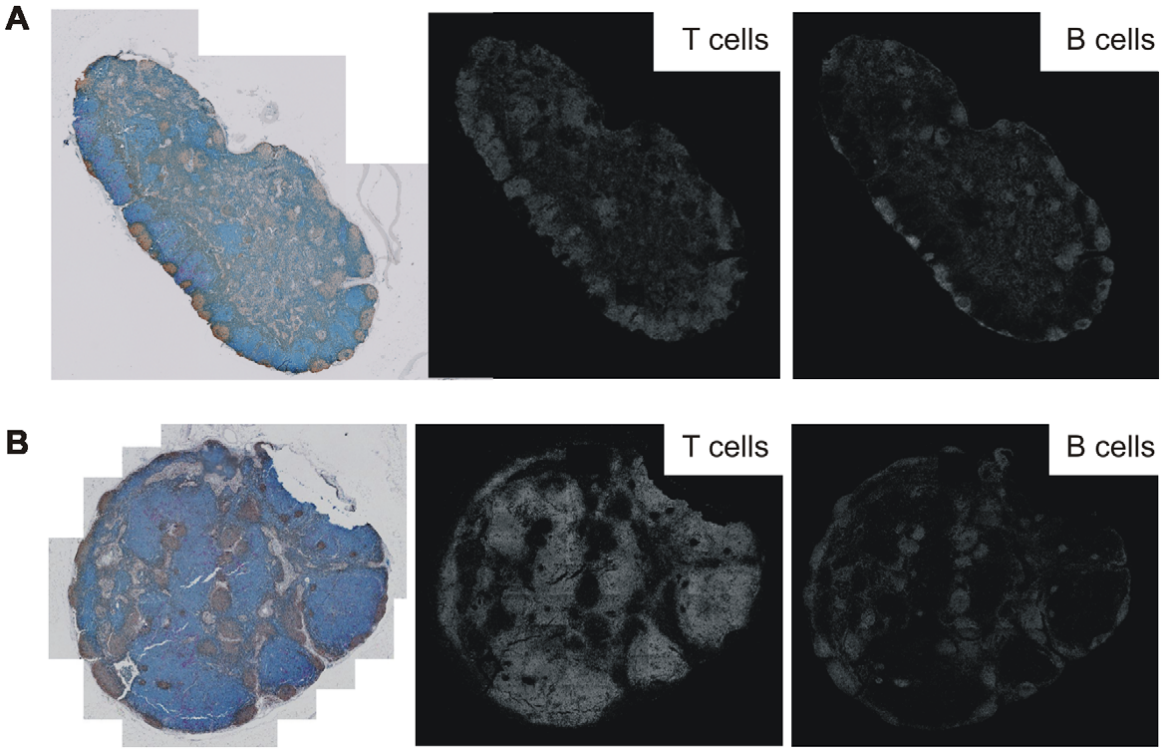
Whole-section images and corresponding maps of T and B cell distributions. (A) A representative cross-section from lymph nodes with similar T and B cell spatial grouping patterns, as indicated by the L function analysis. (B) A representative cross-section from lymph nodes more clustered B than T cells, as indicated by the L function analysis.

We hypothesized that the observed differences in T and B cell grouping patterns as indicated by L functions could be due to different localizations of B cells in TDLNs and HLNs. Thus, we compared the maps of T and B cells that were identified by GemIdent, between lymph node sections with similar T and B cell grouping patterns and those with different grouping patterns. Indeed, we observed that lymph nodes with a similar tendency of the T cells and B cells to clump or disperse appeared to have more B cells in the extrafollicular region compared to lymph nodes with more clustered pattern of B cells (Fig. 4A and B, respectively).

**Figure 5:**
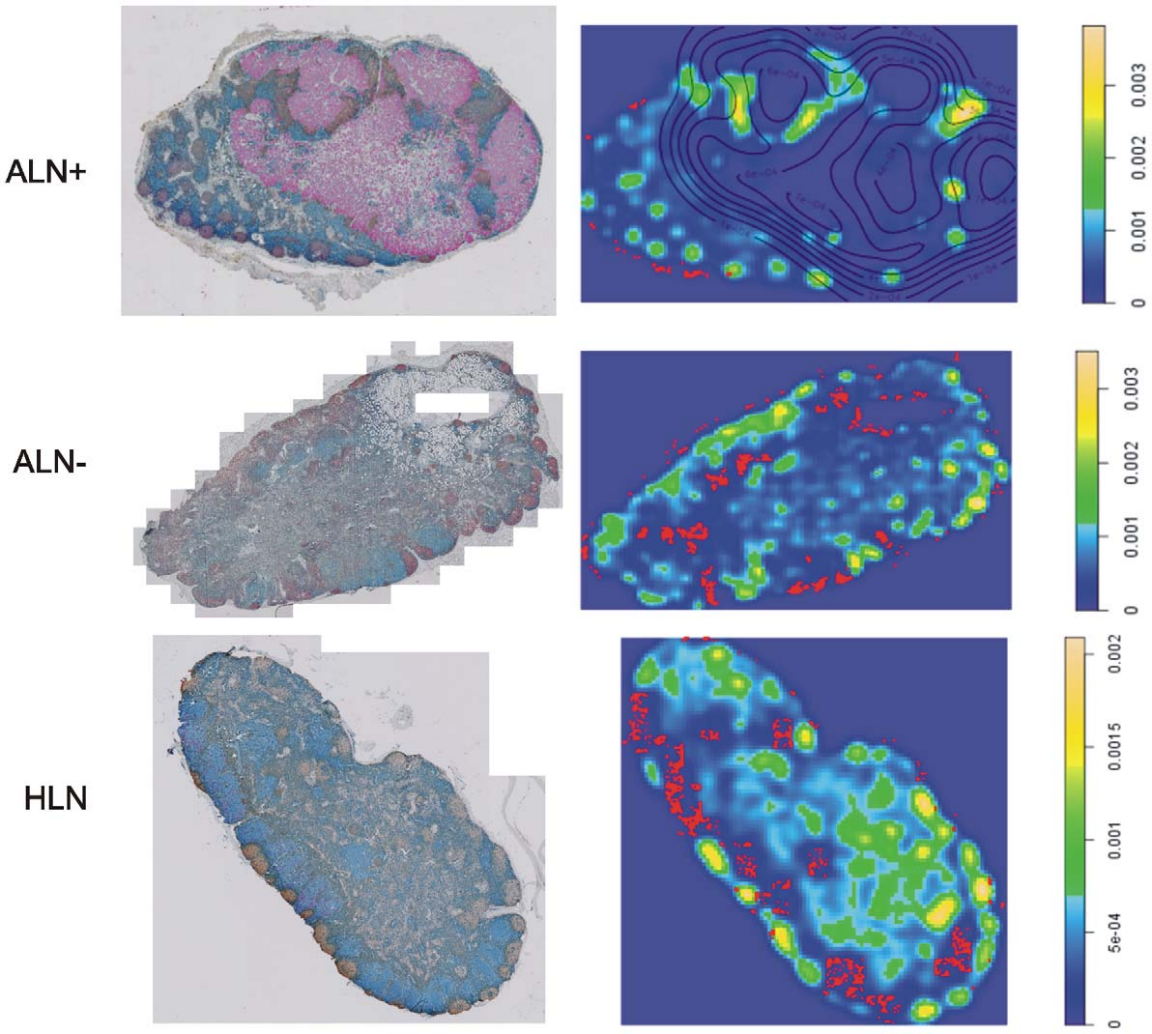
Representative whole-section images and density plots of B cells in an ALN+, ALN− and HLN. The proportion of B cell isolates is the highest in the HLNs. On whole-section images: blue: T cells; brown: B cells; red: tumor cells. On density plots: red dots: B cell isolates (lowest 15% of density); yellow and green: B cell aggregates (highest 85% of density); lines: tumor contours.

We then estimated B cell density on the entire lymph node sections using the Gaussian kernel density estimation method [8]. In all of the lymph nodes analyzed, we found the highest 85^th^ percentile of density (aggregates) mainly inside the follicles (intrafollicular B cells), and the lowest 15^th^ percentile (isolates) outside of the follicles (extrafollicular B cells) (Fig. 5). A comparison of the localization of B cells in tumor-free regions of the TDLNs versus HLNs showed that the average proportion of extrafollicular B cells (±SEM) was significantly lower in the TDLNs: 0.21%±0.11% in ALNs+ (n = 10); 0.26%±0.07% in ALNs− (n = 10); 0.42%±0.09% in SLNs+ (n = 15); and 0.89%±0.19% in HLNs (n = 7); p<0.05 in each of the TDLN group versus the HLN group (t-test). This result confirmed our hypothesis that different localizations of B cells contributed to the dissimilarity in spatial grouping patterns of T and B cells observed in the majority of the TDLNs.

## Discussion

Current pathological examination generates results that are qualitative or semi-quantitative in nature. For example, estrogen and progesterone (ER/PR) receptor expression is generally described by roughly estimating the intensity and percentage of tumor cells staining, such as 3+ (high intensity) and 30%. Within TDLNs, tumor invasion is only distinguished between isolated tumor cells (<0.2 mm), micrometastasis (0.2–2 mm), and metastasis (>2 mm). Even though lymph nodes are immune organs, changes in immune cell populations, such as cell numbers, densities, and spatial relationships, are often unexplored. In this study, we present an integrated approach to quantitatively analyze tissues and capture architectural information. This involves multi-color staining of tissue sections, high-resolution spectral imaging, automated scanning of the entire tissue section, and image analysis to identify each cell, its type and coordinate. A major advantage of multispectral analysis is the ability to unmix different spectra in an image to allow resolution of colors that may appear similar by eye or are overlapping. This enables concurrent identification of different cell phenotypes and functional markers in high complexity staining. By unmixing the overlapping chromogens, one can also observe colocalizing components in the tissue section [9]. This system provides accurate counts of multiple cell types within an entire tissue section, and also cell counts within specific regions - such as B cells within the follicular or paracortex region within a lymph node. Moreover, this approach enables the investigation of spatial relationships between multiple cell types and functional markers within a single tissue section.

As a proof of concept, we tested this integrated approach using SLNs+ from 15 breast cancer patients, pairs of ALN+ and ALN− from 10 patients, and 7 HLNs from prophylactic mastectomy and breast reduction patients. While these selected TDLNs and HLNs have similar proportions of T and B cells, our results revealed different spatial grouping patterns of these cells. In addition, the difference in local T and B cell grouping patterns in tumor-free regions was the highest in tumor-invaded lymph nodes, suggesting that tumor invasion might play a role in enhancing the alteration of T and B cell grouping patterns. These findings illustrate the notion that cell numbers do not capture the full range of information encoded within tissues.

Further investigation of B cell distribution in the TDLNs and HLNs revealed that the proportion of extrafollicular B cells was significantly lower in TDLNs compared to the HLNs. This could contribute to the dissimilarity in spatial grouping patterns of T and B cell observed in the majority of the TDLNs. In lymph nodes that lacked extrafollicular B cells, B cell clustering would appear higher, as they are confined inside follicles as aggregates. Interestingly, a similar trend was observed between the lowest to highest average of B cell isolates found in the extrafollicular, tumor-free regions of various lymph nodes, and the percentages of a certain type of lymph nodes with matching T and B cell grouping patterns: the ALN+ group has the lowest proportion of lymph nodes with matching T and B cell grouping patterns and the lowest average of extrafollicular B cells, followed by the ALN−, SLN+ and HLN groups (Table 1). This correlation further confirms that the lack of B cells in the extrafollicular region of lymph nodes contributed to the appearance of more clustered grouping pattern of B cells compared to the T cells.

**Table 1:** Comparison of the percentages of lymph nodes found with matching spatial grouping patterns of T and B cells and the average proportion of B cell isolates in tumor-free regions.

LN type	% lymph nodes with matching T and B cell grouping patterns	Average % B cell isolates in tumor-free regions (±SEM)
ALN+ (n = 10)	10	0.21±0.11
ALN− (n = 10)	40	0.26±0.07
SLN+ (n = 15)	47	0.42±0.09
HLN (n = 7)	57	0.89±0.19

Upon entry from the bloodstream, T and B cells home to different regions in lymph nodes [10]. Prior to follicular homing and development of antibody responses, extrafollicular localization of B cells is an important event that allows B cells to survey local antigen-carrying APCs and interact with T-helper cells [11]. Reduction of B cells in the extrafollicular T cell zones may lead to ineffective interaction with APCs and impairment of T cell-dependent B cells activation, which can ultimately contributed to defective anti-tumor immune responses.

The results we obtained in this study show that the quantitative image analysis approach is a useful new tool to accurately enumerate different cell populations within the entire tissue section, or within specific regions of interest. Moreover, it can also be applied to investigate alterations in the spatial pattern of various cell populations within tissue sections. This type of study is possible due to multispectral, high-resolution images and analyses of the entire tissue section. Future applications of this quantitative image analysis approach using samples from patients with different disease parameters and outcomes will open up new avenues for investigations on the relationships between cell numbers, architectural patterns, clinical parameters and disease outcomes. This integrated image analysis approach can also be applied to study interactions between cancer and immune cells within tumors, and be extended to study 3-dimensional structures of tumors, lymph nodes, and other organs. In addition to immunohistochemistry, this approach can also be adapted for immunofluorescence and in-situ hybridization techniques. More complex staining combinations using various immune cell markers, as well as their functional markers, are being developed in our laboratory. These preliminary results showed that spatial relationships between immune cells in TDLNs may have an intriguing potential to be developed into novel prognostic tools beyond numerical changes and tumor invasion status.

## Materials and Methods

### Study patients

Twenty five breast cancer patients aged 33–80 years, treated at Stanford University Medical Center in 1997–2004 were evaluated. We analyzed cross sections of SLN+ from 15 patients (1 SLN per patient), and pairs of ALN+ and ALN− from 10 patients. Seven healthy intramammary and axillary lymph nodes were obtained from prophylactic mastectomy and breast reduction patients aged 29–48 years, treated at Stanford in 2001–2007. All samples were obtained from the Stanford pathology archive as coded specimens under a protocol approved by the Stanford University Medical Center Institutional Review Board. The Privacy Notice informed patients that their records might be used for research purposes without their authorization, upon IRB approval. Study procedures are in place to protect the confidentiality of patients' information. Information learned during the study will not affect the treatment of the participants, and thus will not adversely affect their welfare. All subjects were untreated and had no history of cancer or immune disorder prior to their breast cancer diagnosis and SLN biopsy. Following surgical management, patients received adjuvant therapy as determined by their medical and radiation oncologists. Initial diagnosis was performed by needle aspiration or core biopsy in the majority of cases. Final diagnosis was confirmed by the pathologic evaluation of the excision specimen.

### Immunostaining

Three-color IHC staining combinations were performed in order to concurrently visualize B cells (CD20) in brown, T cells (CD3) in blue, and tumor cells (cytokeratin AE1/AE3) in red in lymph node sections. Serial, adjacent 4µm thick tissue sections were cut from formalin-fixed, paraffin-embedded tissue. Prior to IHC staining, antigen retrieval was performed using Diva Decloaker (Biocare Medical, Concord, CA). Primary antibodies used were CD20/CD3 (mouse monoclonal, clone L26/rabbit monoclonal, clone SP7) ready-to-use double stain cocktail and cytokeratin (mouse monoclonal, clone AE1/AE3, 1∶200) to visualize tumor cells. Secondary antibodies used were Mach 2 Double Stain 2 (anti-mouse HRP, anti-rabbit ALP cocktail) and Mach 2 anti-mouse ALP. First, a double staining was performed using the CD20/CD3 cocktail, followed by the secondary antibody Mach 2 Double Stain 2. The chromogens used were DAB for CD20 and Ferangi Blue for CD3. A denaturing solution was used to denature immunoreagents of the first staining sequence. Tumor cells were then identified using the cytokeratin antibody in the subsequent staining, followed by anti-mouse ALP secondary antibody. The cytokeratin was visualized using Vulcan Fast Red chromogen. Optimal concentrations of antibodies and incubation times were optimized in sections of tonsils. All tissue sections were counterstained with hematoxylin. All antibodies and reagents used were obtained from Biocare.

### Imaging

Tissue sections were imaged using a custom-built, high-resolution, automated multispectral imaging system (CRi Vectra™, Woburn, MA). This system can scan entire tissue sections at 200×, yielding sets that consist of hundreds to thousands of high-resolution sub-images in Tagged Image File (TIF) format. The system can also automate the sequential scan of multiple slides. The Vectra™ captures each sub-image at increments of 12 wavelengths between 420 to 720 nm. Using prior examples of each chromogen, it is then able to spectrally un-mix the chromogen signatures into independent channels. This is particularly useful for stains with similar colors that could be confused by eyes, as well as for investigating co-localizations of two or more stains [5].

### Image analysis

The images were loaded into a custom image analysis software, GemIdent, an interactive statistical image segmentation system [5]. The software employs supervised statistical learning. It requires initial training to recognize each cell type and then builds an automatic classifier. The number of training examples required for an accurate classification is dependent on the quality of the staining and the degree of homogeneity in the phenotypes' color and morphological features. This number generally ranges between 50 to 200 training points per phenotype. Run times will vary on the size of the tissue, as well as the complexity of the image. The results include global Cartesian (x, y) coordinates, enumeration of all cells labeled in the section, and maps of all cell populations identified.

### Statistical analysis

The data generated are the coordinates and antibody labels of a large number of points, which represent the nuclei of all cells identified in GemIdent analyses. These form a marked spatial point process that we analyzed using standard spatial statistics [12]. The R statistical packages (spdep and spatstat) were used for the analyses [13], [14].

We used the L function, a variance-stabilized version of the Ripley's K function which allowed us to detect deviations from spatial homogeneity, to analyze the point processes generated from the T and B cells data in each lymph node cross section, and compare the spatial grouping patterns of the T and B cells in tumor-free areas in each of the TDLNs and HLNs. The equation of the K function is as follows:
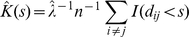
where d_ij_ is the distance between the i^th^ and j^th^ points in a data set of *n* points; λ is the average density of points, generally estimated as *n*/*A*, where *A* is the area of the region containing all points [6].

We randomly selected 800 T cells and B cells from each lymph node section to estimate the L function:
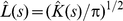
The L function of each cell population is illustrated as a plot, and the plots from the two different cell populations within a tissue section can be compared. From this comparison, we can conclude whether cells in each of the two populations have the same tendency to group or disperse at various distances. A variety of L function plots and their interpretations are illustrated in Figure S4A.

In order to determine the range of interpoint distances to be analyzed in the L function analyses, we computed a distance matrix using 500 B cells that were randomly selected from a lymph node section. From this random sampling, we determined that the minimum distance of any B cell to another was 5.5 µm, and the maximum was 372.5 µm.

Next, we determined the density of cells using Kernel density estimation. This method estimates the density at a point by taking a weighted average of its neighbors. The weighting function is a bivariate Gaussian in this case, making the points that are closer more important than those that are further away [8], [15].

## Supporting Information

Figure S1 An RGB image of an entire TDLN cross section taken by VectraTM. In this particular example, the whole-section image consists of 125, 200× sub-images. Chromogens used were Vulcan Fast Red (cytokeratin (tumor), red), DAB (CD20(+)- B cells, brown) and Ferangi Blue (CD3(+)-T cells, dark blue). Cellular nuclei were counterstained with hematoxylin (light blue).
(3.22 MB TIF)

Figure S2 Spectral unmixing of a triple-stained lymph node section by VectraTM. (A) An original RGB image of a part of a tissue section, taken at 200× magnification. (B) Images resulting from unmixing of the spectral signals of each chromogen and counterstain. (C) A reconstructed image with pseudo-colors that allowed a greater distinction of the cell populations as compared to the original image.
(4.32 MB TIF)

Figure S3 Proportions of both T cells and B cells were similar in TDLNs and HLNs used. (A) Proportions of T and B cells in HLNs and tumor-invaded TDLNs. (B) Proportions T and B cells in ALN+ and ALN− pairs (p = 0.7 and 0.1, respectively; paired t test).
(0.10 MB TIF)

Figure S4 An illustration of L function plots that can be generated from T and B cell data within a tissue section. (A) Interpretations of each plot. (B) An extrapolation of an L function of B cells to another plot that illustrates how much more clustered B cells are compared to the T cells.
(0.36 MB TIF)
