# Supplementary figures and images for: Quantitative, Architectural Analysis of Immune Cell Subsets in Tumor-Draining Lymph Nodes from Breast Cancer Patients and Healthy Lymph Nodes
Source: PLoS One. 2010 Aug 25;5(8):e12420. doi: 10.1371/journal.pone.0012420 (PMC2928294; doi:10.1371/journal.pone.0012420)

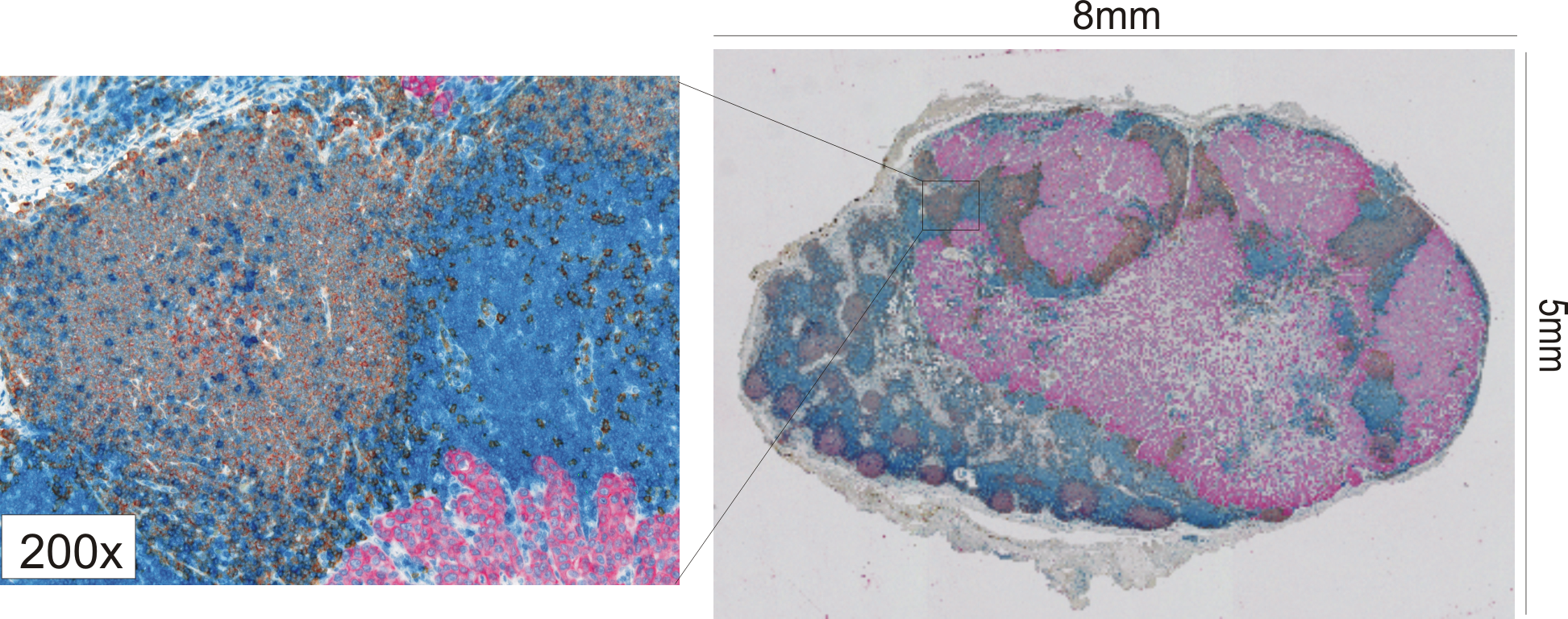

Supplement: Figure S1 — An RGB image of an entire TDLN cross section taken by VectraTM. In this particular example, the whole-section image consists of 125, 200× sub-images. Chromogens used were Vulcan Fast Red (cytokeratin (tumor), red), DAB (CD20(+)- B cells, brown) and Ferangi Blue (CD3(+)-T cells, dark blue). Cellular nuclei were counterstained with hematoxylin (light blue). (3.22 MB TIF) [file pone.0012420.s001.tif]

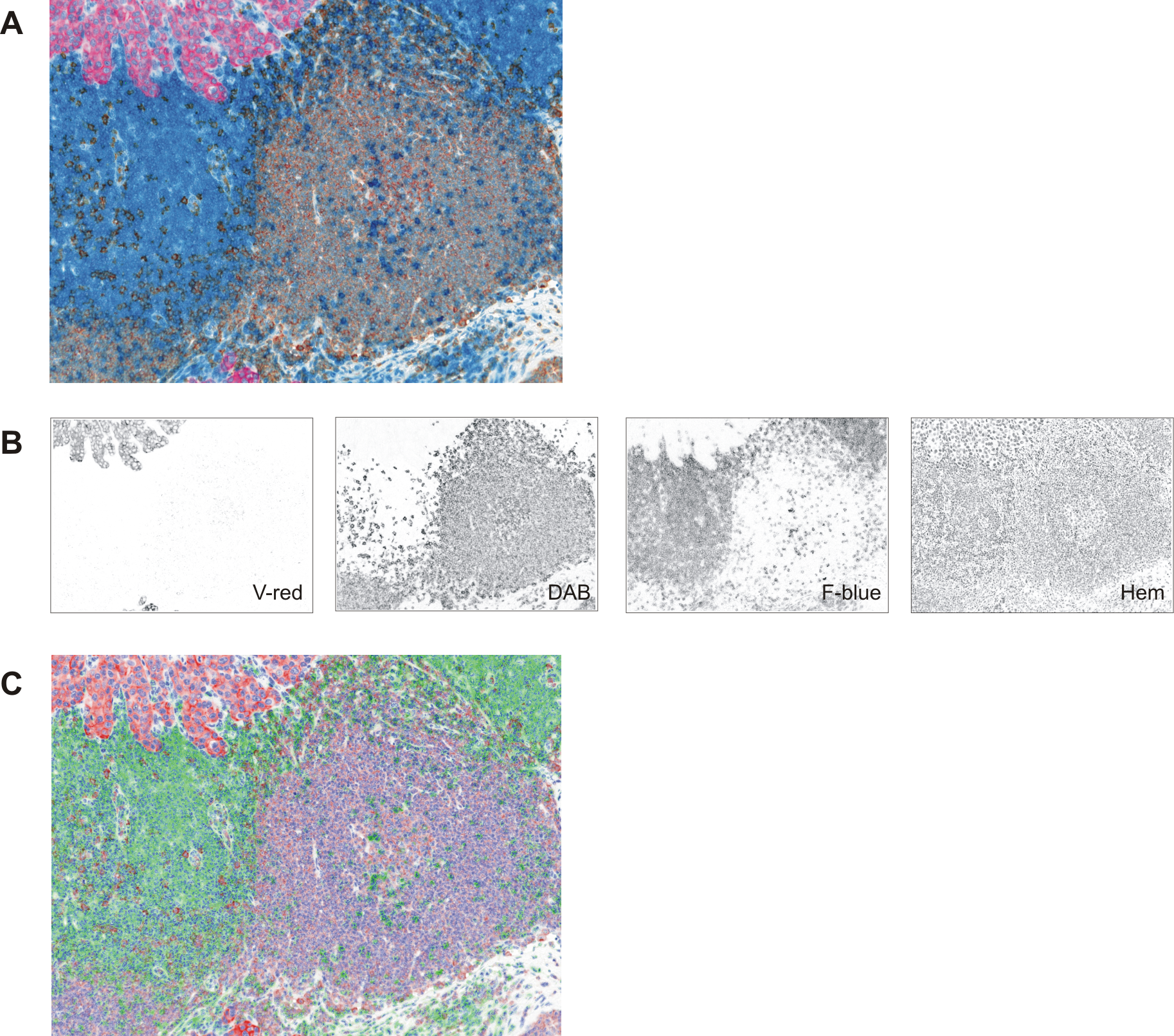

Supplement: Figure S2 — Spectral unmixing of a triple-stained lymph node section by VectraTM. (A) An original RGB image of a part of a tissue section, taken at 200× magnification. (B) Images resulting from unmixing of the spectral signals of each chromogen and counterstain. (C) A reconstructed image with pseudo-colors that allowed a greater distinction of the cell populations as compared to the original image. (4.32 MB TIF) [file pone.0012420.s002.tif]
